# Recombinant Virus-like Particles of Human Parvovirus B19 with the Internal Location of VP1 Unique Region Produced by *Hansenula polymorpha*

**DOI:** 10.3390/v14112410

**Published:** 2022-10-30

**Authors:** Shuai Shao, Qingqing Wang, Yuqin Jin, Xuefeng Zhang, Zhaoming Liu, Shi Chen, Hailan Wu, Sensen Yang, Fang Tang, Jiguo Su, Yu Liang, Jing Zhang, Qiming Li

**Affiliations:** 1 The Sixth Laboratory, National Vaccine and Serum Institute (NVSI), Beijing 101111, China; 2 National Engineering Center for New Vaccine Research, Beijing 101111, China

**Keywords:** parvovirus B19, recombinant vaccine, virus-like particle

## Abstract

Human parvovirus B19 (HPV B19) is pathogenic to human, which can cause fifth disease, transient aplastic crisis, arthritis, myocarditis, autoimmune disorders, hydrops fetalis, and so on. Currently, no approved vaccines or antiviral drugs are available against HPV B19, and thus the development of effective vaccines is needed. The capsid of HPV B19 is composed of two types of proteins, i.e., the major capsid protein VP2 and the minor protein VP1. Previous experimental studies have shown that the dominant immune responses against HPV B19 are elicited by VP1, especially the unique region on the N-terminus of VP1. It has been found that VP2 alone or VP2 and VP1 together can assemble into virus-like particle (VLP). The VLP structure formed by VP2 has been resolved, however, the location of VP1 in the capsid, especially the location of VP1 unique region with strong immunogenicity, is still not clear. In the present work, using the *Hansenula polymorpha* expression system developed by our laboratory, two kinds of recombinant HPV B19 VLPs were expressed, i.e., the VLP co-assembled by VP1 and VP2 (VP1/VP2 VLP) and the VLP whose VP1 content was improved (VP1h/VP2 VLP). The expression, purity, and morphology of these two VLPs were characterized, and then their immunogenic properties were investigated and compared with those of the VLP containing VP2 alone (VP2 VLP) previously developed by our group. Furthermore, the location of the VP1 unique region in the VLPs was determined by using the immunogold electron microscopy (IGEM). Our experimental results show that the VP1h/VP2 VLP elicits a stronger neutralization against the HPV B19 than VP2 and VP1/VP2 VLPs, which implies that the increase of VP1 content significantly improves the level of neutralizing antibodies. In addition, the IGEM observations suggest that the unique region of VP1 may be located inside the recombinant VLP. The VLPs recombinantly expressed by our *Hansenula polymorpha* system may serve as a promising candidate immunogen for HPV B19 vaccine development.

## 1. Introduction

Human parvovirus B19 (HPV B19) was first discovered in the sera of blood-donors and patients by Cossart and his colleagues in 1975 [1,2]. The subsequent studies found that HPV B19 was the pathogen responsible for many diseases including transient aplastic crisis [3], fifth disease [4], arthropathy [5], myocarditis [6], autoimmune disorders [7], hydrops fetalis [8], and so on. The epidemiological investigations have shown that HPV B19 is highly infective and prevalent worldwide, and it is estimated that approximately 85% persons have been infected by HPV B19 at some point in their lives [9]. For most healthy individuals with competent immunity, the infection of HPV B19 can elicit neutralizing antibodies to clear the virus and establish permanent immune protections against the virus. However, the infection of HPV B19 can result in serious consequences in the immunocompromised persons and the fetus [10,11]. At present, no antiviral drug is available for the treatment of HPV B19 infection, and therefore the development of effective vaccines against the virus is important.

HPV B19 is a nonenveloped, single-stranded linear DNA virus, as a member of *Erythrovirus* genus of *Parvoviridae* family [12]. HPV B19 virus particle has an icosahedral symmetric structure assembled by 60 capsid subunits, and these subunits include two types of capsid proteins: the major capsid protein VP2 and the minor capsid protein VP1. In the wild-type HPV B19 virus particle, VP2 constitutes approximately 95% of the viral capsid and the remaining 5% is VP1. VP1 and VP2 are translated from the same open reading frame with different splicing patterns. The C-terminal of VP1 shares the same sequence with VP2, and besides that, VP1 contains additional 227 amino acids in its N-terminal region called VP1 unique region (VP1u) [13]. 

HPV B19 cannot yet be cultured in vitro, and the production of recombinant virus-like particles (VLPs) is a promising strategy for the development of effective vaccines against the virus. Several experimental studies have shown that either VP2 alone or VP2 and VP1 together can form VLPs, but VP1 alone cannot self-assemble into VLPs. Although VP2 is necessary for the formation of VLPs, VP1 plays critical roles in eliciting the neutralizing immune responses against HPV B19 and many neutralizing antigenic epitopes have been found to be located on VP1u [14,15,16]. Kajigaya et al. produced the recombinant HPV B19 VLPs containing VP2 and VP1 by Sf9 cells, and they found that the VLPs composed of VP2 and VP1 could elicit a neutralizing antibody response [17]. Furthermore, Bansal et al. constructed HPV B19 VLPs with various contents of VP1, and the immunological experiments showed that the increasing of VP1 content significantly improved the elicitation of neutralizing antibodies. The VLPs with ≥25% VP1 contents effectively induced persistent neutralizing immune response against HPV B19 [18,19]. The above experimental results indicate that the amounts of VP1, especially VP1u, are important for the neutralizing immunogenicity of HPV B19 VLPs. These recombinant two-component VLPs were expressed by the co-infecting insect cell (Sf9) with double baculoviruses in which one baculoviruse expresses VP2 and the other expresses VP1 [19,20]. Besides that, Chandramouli et al. produced two-component VLPs by dual expressions of VP2 and VP1 in a single plasmid in *Saccharomyces cerevisiae*, and a new promoter was constructed to regulate the expression ratio of VP1 to VP2 [20]. Suzuki et al. constructed two plasmids encoding VP1 and VP2, respectively, which were mixed (1:1) and transfected into 293 T cells to obtain two-component HPV B19 VLPs with a VP1:VP2 ratio of approximately 50:50 [21]. 

The three-dimensional structure of HPV B19 VLP formed by VP2 alone has been determined by X-ray crystallography with the resolution of 3.5 Å [13]. However, the location of VP1, especially VP1u with strong immunogenicity, is still not clear, and seemingly conflicting results were obtained by several previous experimental investigations. The tagged monoclonal antibody-binding assays revealed that VP1u is exposed on the surface of the capsid for the recombinant HPV B19 VLPs expressed by the insect cell (Sf9) with baculoviruses [17,22]. While for the natural capsid of HPV B19, the location of VP1u was determined, also by using monoclonal antibody-binding assays, to be internal [23]. Only after the attachment of the receptor, increasing the temperature or reducing PH value, the externalization of VP1u occurred [23,24]. It was suggested that VP1u adopted different locations and conformations between the natural viral particle and the VLP expressed by the insect cell. 

In the present work, using the *Hansenula polymorpha* expression system developed by our laboratory, VP1 and VP2 were co-expressed to produce the two-component HPV B19 VLP. Furthermore, a stronger expression promoter was adopted to improve the relative proportion of VP1 in the VLP. In our study, two types of VLPs were produced, i.e., the VLP co-assembled by VP1 and VP2 (VP1/VP2 VLP) and the VLP with an improved VP1 content (VP1h/VP2 VLP). The expression, purity, and morphology of these two VLPs were characterized, and then their immunogenicity was evaluated and compared with that of the VLP formed by VP2 alone (VP2 VLP). In addition, the location of the immunodominant VP1u in the recombinant VLPs was determined by using the immunogold electron microscopy (IGEM).

## 2. Materials and Methods

### 2.1. HPV B19 VLP Plasmid Construction

**Figure 1:**
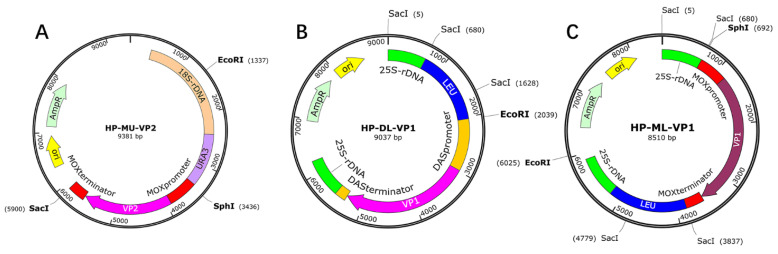
Schematic structures of the constructed VP2, VP1 and VP1h plasmids. (**A**): The constructed VP2 plasmid (HP-MU). (**B**): The constructed VP1 plasmid (HP-DL). (**C**): The constructed VP1h plasmid (HP-ML).

In the present study, two recombinant HPV B19 VLPs were produced, i.e., VP1/VP2 and VP1h/VP2 VLPs. For the expression of these two-component VLPs, the plasmids for VP2 and VP1 were constructed, respectively. In the VP2 plasmid construction, the gene codons of HPV B19 VP2, which was truncated from the VP1 gene (Genbank accession No. AY386330.1), were codon-optimized and then cloned into the “HP-MU” shuttle plasmid constructed by our laboratory. This plasmid contains a URA3 selectable marker, a MOX promoter and terminator, as well as a 18S rDNA homologous recombination arm responsible for the integration of the target gene into the yeast genome [25], as shown in Figure 1A. In this plasmid, the VP2 gene was inserted between the MOX promoter and terminator. Different from VP2 plasmid, in the VP1 plasmid construction, the “HP-DL” shuttle plasmid was used, which includes a LEU2 selectable marker, a DAS promoter and terminator, and a 25S rDNA homologous recombinant arm (Figure 1B). The VP1 gene was inserted between the DAS promoter and terminator. Then, the VP1 and VP2 double plasmids were linearized and transformed by electroporation into the URA3/LEU2 auxotrophic *Hansenula polymorpha* (named NVSI-H.P-105, ΔURA3ΔLEU2) developed by our laboratory in which the key gene elements of the synthesis of uracil and leucine were deleted by UV irradiation and genetic engineering methods [26] to co-express the VP1/VP2 VLP. 

Furthermore, in order to improve the expression level of VP1 to generate the VP1h/VP2 VLP, the DAS promoter and terminator in the VP1 plasmid were replaced by a stronger MOX promoter and terminator to construct the VP1h plasmid called “HP-ML” in which the VP1 gene was inserted between the MOX promoter and terminator (Figure 1C). Then, the VP1h plasmid was co-expressed with VP2 plasmid by NVSI-H.P-105 *Hansenula polymorpha* to produce VP1h/VP2 VLP.

### 2.2. HPV B19 VLP Expression and Purification

The *Hansenula polymorph* yeast strains with high-level expressions were selected to produce the recombinant VLPs by high-density fermentation. In our study, to select the *Hansenula polymorph* strains with high-level protein expressions, the plasmid vector encoding VP2 was firstly linearized and transformed by electroporation into the *Hansenula polymorpha*, and the strains with high expression of VP2 were screened. Then, the plasmid encoding VP1 or VP1h was also transformed into the cells to realize the co-expression of VP1 and VP2, and the highly expressing strain was finally selected to produce the two-component VLPs. During the strain screening process, the VP2 protein expression level was evaluated by enzyme linked immunosorbent assay (ELISA) using the VP2-binding antibody MAB8293 (purchased from Millipore Co.) as the detection antibody, and the VP1 expression level was determined using the VP1u-specific polyclonal antibodies (PcAbs) obtained by using VP1u to immunize rabbits. The strain with the highest optical density (OD) value at 450/630 nm in the ELISA assay was selected as the high-expression strain.

For the high-density fermentation of the *Hansenula polymorpha*, the highly expressing strain was inoculated in 20 mL MD medium (13.4 g/L yeast nitrogen base w/o amino acids, 2% glycerin). After incubation at 37 °C and 230 rpm for 24 h, it was transferred to 100 mL MD medium and incubated at 37 °C and 230 rpm for an additional 24 h. After amplification, 100 mL of the *Hansenula polymorpha* solution was added into the bioreactor (Bioengineering AG KLF2000-3) containing yeast culture medium. The temperature was set to 37 °C, and the stirring speed gradually increased to 800 rpm with 100% aeration. After incubation for approximately 24 h and the rise of the dissolved oxygen to more than 100%, the feed pump was opened for glucose de-repression so that the dissolved oxygen in the reactor was maintained at approximately 60%. After glucose de-repression for 24 h, methanol was added to trigger the expression of the target protein, and the yeast solution was collected after continuous induction culture for 72 h.

The recombinant VLPs were purified using the gel column chromatography (GE Healthcare Sephacryl S-500 column) followed by the anionic-exchange chromatography (GE Healthcare Capto Q column). Specifically, during the purification of the recombinant VLPs, the *Hansenula polymorpha* cells was crushed by high pressure cracker in the buffer solution (50 mM PB, 200 mM NaCl, pH8.0), and the supernatant was separated by centrifugation. Then, the supernatant sample was roughly purified by two-aqueous phase extraction, followed by the purification using the gel column chromatography in the buffer solution (50 mM PB, 200 mM NaCl, pH 7.4). The collected protein sample was further purified using the anionic-exchange chromatography in the buffer solution (25 mM PB, 100 mM NaCl, pH7.4). Subsequently, the purified VLPs were obtained by elution with the buffer solution (25 mM PB, 1.5 M NaCl, pH7.4).

### 2.3. HPV B19 VLP Characterization

The purified recombinant HPV B19 VLPs were analyzed by SDS-PAGE and Western blot to verify the expression of VP1 and VP2 proteins. In Western blot experiments, a monoclonal antibody (mAb) (anti-parvovirus B19 5E408 purchased from Santa Cruz Biotechnology, Inc.), which can bind to both VP1 and VP2, was used to detect the presence of VP1 and VP2, and the polyclonal antibodies (PcAbs) specific to VP1u (produced by our laboratory) were also applied to detect the presence of VP1. The purity of the VLPs was measured by using the size exclusion chromatography combined with the high-performance liquid chromatography (SEC-HPLC) method. The relative contents of VP1 and VP2 in the recombinant VLPs were determined by SDS-PAGE combined with the gray scanning software ImageQuant TL. 

The integrity, homogeneity and morphology of the recombinant VLPs were examined by transmission electron microscopy (TEM). In TEM, the VLP samples were dropped onto the carbon-coated copper grids. After the absorption for 5 min, the samples were negatively stained with 2% phosphotungstic acid solution and subsequently observed by TEM (FEI Tecnai 12).

### 2.4. The Immunogold Electron Microscopy Experiment

The immunogold electron microscopy (IGEM) was utilized to determine whether the VP1u is located inside or outside of the capsid in the VP1/VP2 and VP1h/VP2 VLPs. The VP1u-specific PcAbs were used to detect the exposed VP1u in the VLP samples. If VP1u is located in the interior of the capsid, the PcAbs cannot bind to the VLP, whereas if VP1u is exposed on the surface of the capsid, the PcAbs can attach to the VLP and can then be detected by IGEM. 

In the experiment, the VLPs were firstly absorbed on the sample carrier of the TEM and blocked with 1% bovine serum albumin (BSA) for 10 min, which then floated on the droplets of VP1u-specific PcAbs. After incubation at room temperature for 2 h, the carrier was washed three times with 1% BSA/Phosphate Buffered Saline (PBS) for 10 min. Subsequently, the carrier floated on the droplets of the secondary antibody labeled by colloidal gold and incubated at room temperature for 1 h. After washing three times with 1% BSA/PBS for 10 min, the carrier was placed on the droplets of 0.5% glutaraldehyde for 10 min. Then, the carrier was washed three times by ultrapure water and negatively stained with phosphotungstic acid. After dyeing, the carrier was dried and observed by TEM (FEI Tecnai 12). 

### 2.5. Mice Immunization

The expressed HPV B19 VP1/VP2 and VP1h/VP2 VLPs, along with the VP2 VLP produced previously by our group [25], were respectively adsorbed by aluminum adjuvant. Then, for each VLP, 6 BALB/c female mice (purchased from Beijing Vital River Laboratory Animal Technology Co., Ltd.) with 6–8 weeks old were immunized intraperitoneally. A total of three doses, each of 0.5 mL containing 0.5 μg antigen and 0.3 mg aluminum adjuvant, were administered to the mice with the intervals of 2 weeks. Another six mice were injected in the same way only with adjuvant, serving as the control group. Two weeks after the completion of the immunizations, blood was drawn from these immunized mice for immunogenicity evaluation. 

### 2.6. Enzyme Linked Immunosorbent Assay to Evaluate the IgG Antibody Level

The titer of IgG antibodies in the sera of the immunized mice were evaluated by enzyme linked immunosorbent assay (ELISA) using the VP2 VLP to coat the plate. The purified VP2 VLP produced in our previous study [25] were diluted to 4 μg/mL, which were then coated onto the plates with 100 μL per well at 2–8 °C overnight. The plates were blocked with PBS and BSA at 37 °C for 3 h, followed by washing three times with PBST (KH_2_PO_4_, Na_2_HPO_4_·12H_2_O, NaCl, KCl, Tween-20) buffer. The serum samples from the immunized mice were firstly diluted to 1:200, which is followed by 5-fold serial dilutions. The diluted sera were added into the well with 100 μL per well and incubated at 37 °C for 1 h. After washing the plates three times with PBST, the secondary antibody, i.e., HRP-labeled goat anti-mouse IgG (purchased from ZSGB-Bio), at the dilution of 1:16,000 was added into the well with 100 μL per well, which continued to be incubated at 37 °C for 1 h. Subsequently, the plate was washed three times with PBST, and 50 µL tetramethylbenzidine and 50 µL hydrogen peroxide solutions were added to the well. After color development for 5 min, the reaction stop solution (0.2 M sulfuric acid) was added into the well with 50 μL per well, and the value of optical density at 450/630 nm (OD_450/630nm_) was obtained from the plate reader. The OD_450/630nm_ value measured for the well without the addition of serum was taken as the blank control, and the cutoff value of OD_450/630nm_ was set to be 2.5 times of the blank value. The titer of IgG antibodies was determined as the reciprocal of the maximum dilution of the serum whose OD_450/630nm_ is equal to or greater than the cutoff value.

### 2.7. Hemagglutination Inhibition Assay to Evaluate the Neutralizing Antibody Level

Due to the fact that HPV B19 cannot be cultured in vitro, the serum neutralizing antibodies cannot be directly evaluated by microneutralization assay. The P-antigen (globoside) on the membrane of human red blood cells is the receptor for HPV B19 virus, and HPV B19 VLPs can specifically bind to the receptor, which causes the agglutination of the red blood cells (RBCs) [27,28,29,30]. This phenomenon provided an effective method to measure the titer of the neutralizing antibodies through evaluating the anti-hemagglutination activity of the serum. The hemagglutination inhibition (HI) assay has been largely used in detecting the neutralizing antibodies to many infective viruses [31]. 

Before the HI assay, we first detected the hemagglutination of RBCs induced by the HPV B19 VLPs in which the recombinant VP1/VP2 VLP was used. In this experiment, 4 mL fresh human blood was mixed with 4 mL Alsever’s solution, which was subsequently centrifuged at 1000*g* under 2–8 °C for 10 min. The supernatant was abandoned and the RBCs were collected. The RBCs were then washed three times with PBS-BSA buffer (0.01 M PB, 0.9% NaCl (*w*/*v*), 0.1% BSA(*w*/*v*)). The washed RBCs were diluted by PBS-BSA buffer to produce the RBCs suspension with the concentration of 50%, which was then stored at 2–8 °C for the following experiments. The VP1/VP2 VLP sample with a starting concentration of 100 μg/mL was diluted by 2-fold serial dilutions and added into the well of the U-type 96-well plate. Another well was added by PBS-BSA buffer as a control. Subsequently, 1% RBCs suspension produced above was added into the wells to observe the hemagglutination of the RBCs. Base on this RBCs hemagglutination experiment, the optimal VLP concentration causing the hemagglutination of the RBCs was determined to be 12.5 μg/mL, which was then used in the following HI assays.

In the HI assay, the serum samples from the immunized mice were diluted by 2-fold serial dilutions starting with 1:10 and the diluted sera were added into the wells of the plate. Then, 25 μL VP1/VP2 VLPs with the concentration of 12.5 μg/mL were added into the wells and incubated with the sera at room temperature for 1 h. Subsequently, 1% RBC solution was added into the wells and incubated at 4 °C for 2 h. Finally, the inhibition of the hemagglutination of the RBCs was observed, and the titer of HI antibodies was evaluated as the reciprocal of the maximum dilution of the sera that completely inhibits RBCs hemagglutination. 

## 3. Results

### 3.1. The Production and Characterization of the Recombinant HPV B19 VLPs by Hansenula polymorpha

**Figure 2:**
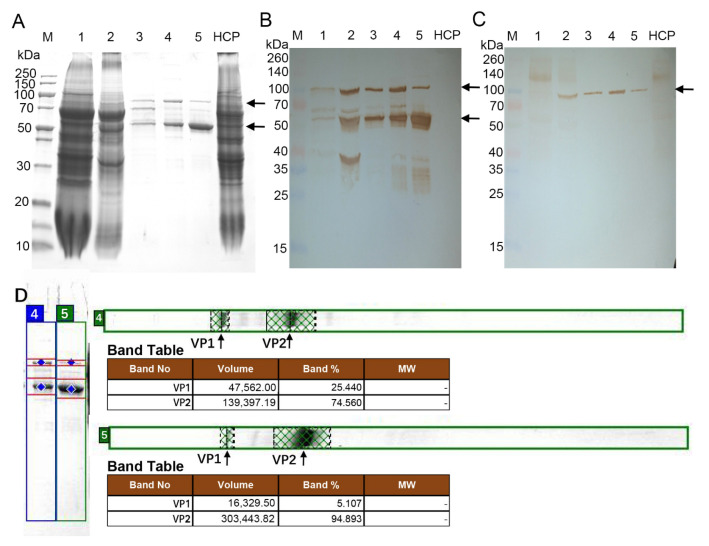
SDS-PAGE and Western blot analysis results of the VP1/VP2 and VP1h/VP2 VLPs recombinantly expressed by using the *Hansenula polymorpha* yeast. (**A**): SDS-PAGE of the VP1/VP2 and VP1h/VP2 VLPs. (**B**): Western blot analysis of the VP1/VP2 and VP1h/VP2 VLPs in which the anti-parvovirus B19 5E408 monoclonal antibody was used. (**C**): Western blot analysis of the VP1/VP2 and VP1h/VP2 VLPs in which the anti-VP1u polyclonal antibodies were used. (**D**): Gray scanning results for the relative contents of VP1 and VP2 in the VP1/VP2 and VP1h/VP2 VLPs by using the ImageQuant TL software. M: marker; Lane 1: whole-cell lysate proteins of the *Hansenula polymorpha* expressing VP1h/VP2 VLP; Lane 2: VP1h/VP2 VLP sample after crude purification by two-aqueous phase extraction; Lane 3: VP1h/VP2 VLP sample after purification using the gel column chromatography; Lane 4: VP1h/VP2 VLP after further purification using the anionic-exchange chromatography; Lane 5: VP1/VP2 VLP after gel column chromatography and anionic-exchange chromatography purifications; HCP: host cell proteins. The bands corresponding to the VP1 and VP2 proteins were marked by black arrows.

In this study, the recombinant B19 VLP co-assembled by VP1 and VP2 (VP1/VP2 VLP) was expressed by the URA3/LEU2 auxotrophic *Hansenula polymorpha* (NVSI-H.P-105, ΔURA3ΔLEU2) developed by our laboratory. The expressed VLP was purified by the gel column chromatography and the anionic-exchange chromatography. Then, the SDS-PAGE and Western blot experiments were carried out to detect the expression of the component proteins of the recombinant VP1/VP2 VLP. The experiment results are shown in Figure 2. Two specific bands with the molecular weights of 58 kDa and 90 kDa can be detected (Figure 2A), which corresponds to the theoretical molecular weights of VP2 and VP1, respectively. This result demonstrates the presence of both VP1 and VP2 in the VLP. Western blot experiments show that both VP1 and VP2 can specifically bind to the anti-parvovirus B19 mAb (Figure 2B). These results confirm the successful expression of VP1 and VP2 in the recombinant VP1/VP2 VLP.

It has been revealed that VP1, especially VP1u, plays important roles in mediating the attachment of HPV B19 to the host cells, and many immunodominant neutralizing epitopes have been identified to be located at VP1u [14,15,16]. Several experimental studies have indicated that improving the amount of VP1 in the VLP can distinctly increase the elicitation of neutralizing antibodies [18,19]. In the present study, the DAS promoter in the VP1 plasmid was replaced by a stronger MOX promoter to enhance the proportion of VP1 in the recombinant VLP. Using the optimized plasmid, the recombinant VP1h/VP2 VLP was produced in which the proportion of VP1 was highly improved. The expression of VP1h/VP2 VLP was also detected by SDS-PAGE and Western blot. The specific bands corresponding to VP1 and VP2 can be seen, as shown in Figure 2A, and the molecular weights determined experimentally agree with the theoretical values. The Western blot experiments show that both VP1 and VP2 specifically bind with the anti-parvovirus B19 mAb (Figure 2B), but only VP1 specifically binds with the anti-VP1u PcAbs (Figure 2C).

The amount of VP1 in the two recombinant VLPs, i.e., VP1/VP2 and VP1h/VP2 VLPs, was determined, respectively, by SDS-PAGE combined with the gray scanning software ImageQuant TL. In the VP1/VP2 VLP, the proportion of VP1 was measured to be approximately 5.11%, where the value is consistent with that of the natural HPV B19 virus [23]. Whereas, in the recombinant VP1h/VP2 VLP, the proportion of VP1 was evaluated to be approximately 25.44%, which is significantly improved compared with that in VP1/VP2 VLP (Figure 2D).

**Figure 3:**
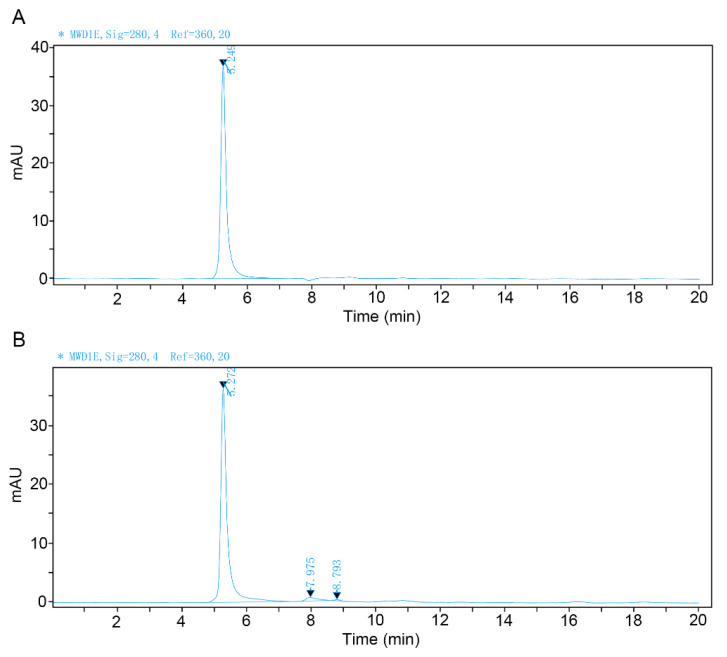
SEC-HPLC analysis results of the recombinant VP1/VP2 and VP1h/VP2 VLPs after purification. (**A**): SEC-HPLC analysis of the VP1/VP2 VLP. (**B**): SEC-HPLC analysis of the VP1h/VP2 VLP.

The purity of these two recombinant VLPs, i.e., VP1/VP2 and VP1h/VP2 VLPs, was analyzed by the SEC-HPLC method. The experimental results are shown in Figure 3. It was found that the purity of both VLPs is higher than 95%.

**Figure 4:**
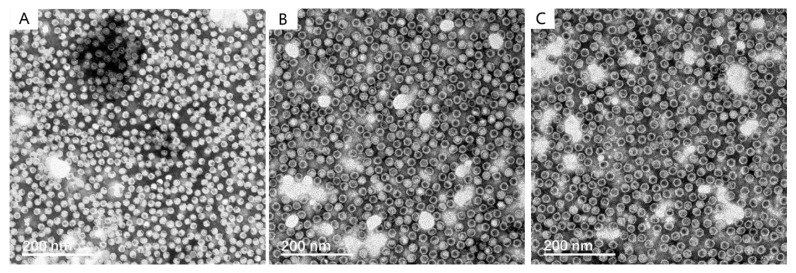
Morphological observation of the recombinant VP1/VP2 and VP1h/VP2 VLPs by using negative staining transmission electron microscopy (TEM), which was compared with that of the VP2 VLP produced by using the same *Hansenula polymorpha* expression system in our previous study [25]. (**A**): The TEM micrograph of the VP2 VLPs. This TEM graph was taken by the same sample but in a slightly different scope, with permission from Ref. [25] published by Chinese Journal of Microbiology and Immunology, 2020. (**B**): The TEM micrograph of the VP1/VP2 VLPs. (**C**): the TEM micrograph of the VP1h/VP2 VLPs.

The integrity, homogeneity and morphology of the recombinant VLPs were observed by negative staining TEM, which were compared with those of the VP2 VLP produced by using the same *Hansenula polymorpha* expression system in our previous study [25]. The TEM observations show that the particle size is uniform and the shape is regular both for VP1/VP2 and VP1h/VP2 VLPs, which is similar to those of VP2 VLP, as shown in Figure 4. The diameter of the VLP is approximately 20 nm, and there are no obvious differences among these three types of recombinant VLPs. 

### 3.2. Evaluation of the Immunological Effects of the Recombinant VP1/VP2 and VP1h/VP2 VLPs

**Figure 5:**
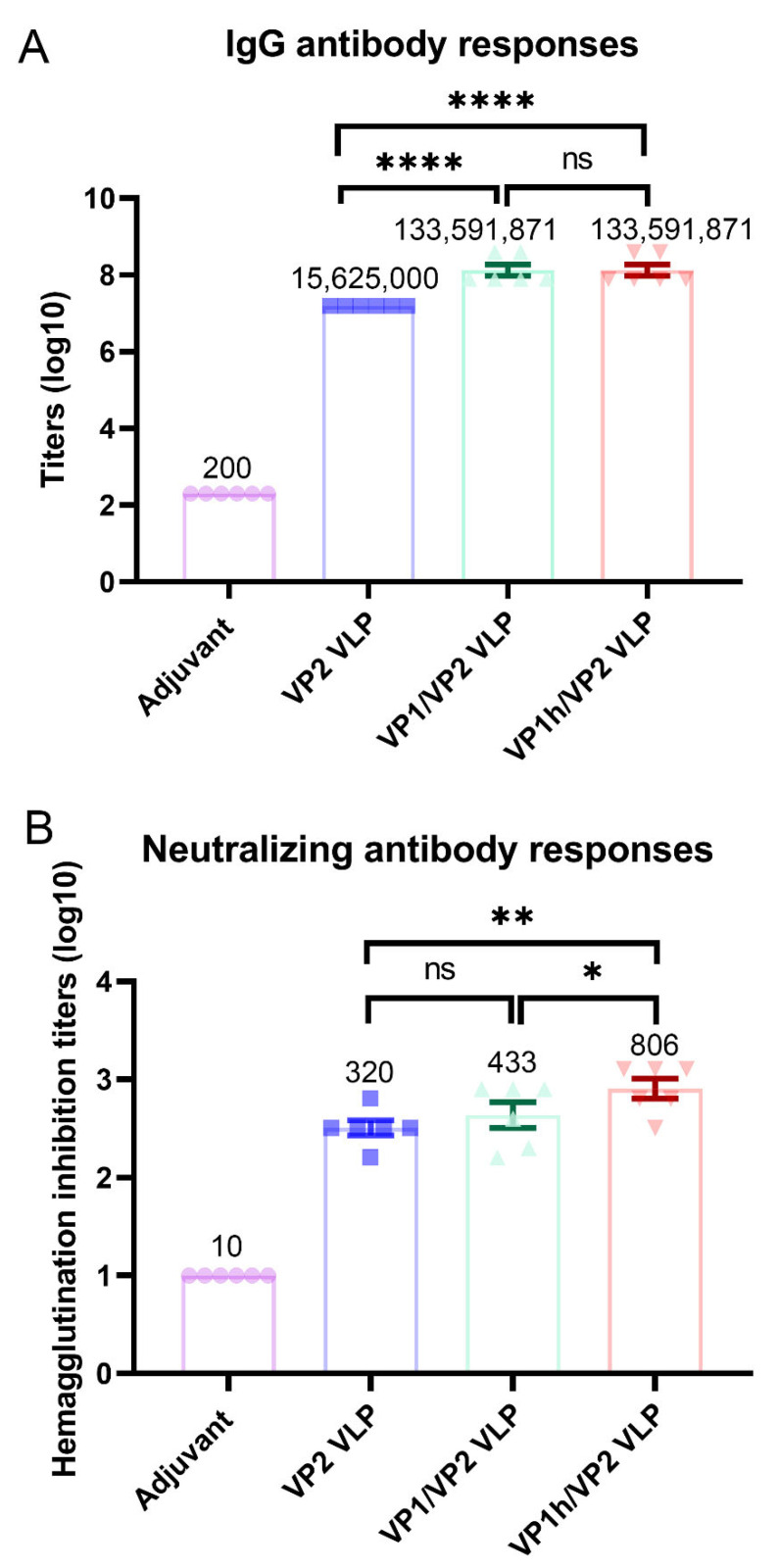
Immune responses induced by the recombinant VP1/VP2 and VP1h/VP2 VLPs compared with that induced by the VP2 VLP. (**A**): IgG antibody levels evaluated by enzyme linked immunosorbent assay. (**B**): Neutralizing antibody levels evaluated by hemagglutination inhibition assay. Data are presented as mean ± SEM. GMT values are given on the top of each bar of the histogram. One-way ANOVA followed by the LSD *t*-test was applied to detect statistical difference between groups. * *p* < 0.05, ** *p* < 0.01, **** *p* < 0.0001, ns: not significant.

The immunogenicity of the recombinant VP1/VP2 and VP1h/VP2 VLPs was evaluated and compared with that of the VP2 VLP produced previously by our laboratory using the same expression system. For each recombinant VLP, 6 BALB/c female mice were immunized and the serum was collected. Then, the titer of IgG antibodies in the sera was measured by using ELISA, and furthermore the titer of neutralizing antibodies was evaluated by using HI assay. The ELISA results show that both the VP1/VP2 and VP1h/VP2 VLPs elicit a high level of IgG antibodies, as displayed in Figure 5A. There is no statistical difference in the geometric mean titers (GMTs) of IgG antibodies between these two recombinant VLPs. However, the IgG antibody GMTs induced by the VP1/VP2 and VP1/VP2 VLPs are significantly higher than that elicited by the VP2 VLP, as shown in Figure 5A, which demonstrates that VP1 plays an important role for the immunogenicity of the recombinant VLPs.

Then, the levels of neutralizing antibodies in the sera from the mice immunized with different VLPs were evaluated by using HI assays, and the detection results are displayed in Figure 5B. It is found that the GMT of neutralizing antibodies elicited by VP2 VLP is 320, whereas those induced by VP1/VP2 and VP1h/VP2 were improved to 433 and 806, respectively. Importantly, the neutralizing antibody level induced by VP1h/VP2 VLP is significantly higher than those by VP2 and VP1/VP2 VLPs, indicating that the increase of VP1 content can significantly improve the neutralizing activity of the recombinant VLPs. Our results are consistent with the studies of other groups [18,19]. The containing of VP1 component and increasing its content may be necessary for the development of the vaccines against HPV B19.

### 3.3. Whether or Not VP1u Exposed on the VLP Surface Detected by Anti-VP1u PcAb Combined with Immunogold Electron Microscopy

**Figure 6:**
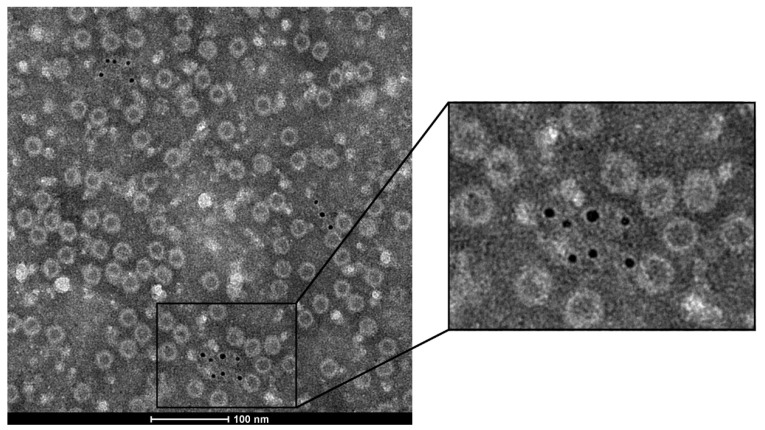
The immunogold electron microscopy (IGEM) observation of the binding of the VP1u-specific polyclonal antibodies with the VP1h/VP2 VLPs to determine whether the VP1u is located inside or outside of the capsid.

The above experimental results demonstrate that VP1u plays an important role in the immune activity of the recombinant HPV B19 VLPs. However, the location of VP1u in the capsid structure is still not clear, and two seemingly conflicting views have been proposed. For the recombinant VLPs expressed by the insect cell, VP1u was detected to be exposed on the surface of the capsid [17,22], whereas in the natural HPV B19 virus VP1u was determined to be located inside the capsid [23]. In this study, whether VP1u is exposed in our recombinant VLPs was detected by using the anti-VP1u PcAbs combined with IGEM. The visualized results by IGEM for VP1h/VP2 VLP are displayed in Figure 6. It is found that for the recombinant VLPs, the immunogolds were only detected around the non-integrated capsid particles. However, around the integrated particles, the immunogold was not found. These results indicate that VP1u may be embedded inside the capsid, which is not accessible to the anti-VP1u PcAbs in the integrated VLPs. Only when the VLP was broken and the VP1u was exposed, can the anti-VP1u PcAbs bind to it and be detected by IGEM. The IGEM observation results suggested that VP1u may be located inside the capsid for our recombinant two-component VLPs expressed by *Hansenula polymorpha*. 

## 4. Discussion

As appropriate culture systems are still not available for HPV B19 infection, the recombinant VLPs are considered promising candidates for developing vaccines against the virus. Our experimental results and several previous studies have demonstrated that the containing of VP1 component in the VLP and improving its amount to a certain level are critical for eliciting a high neutralizing antibody response [17,18,19,20,21]. However, the co-expression of VP1 and VP2, as well as the control of their relative ratio, with high productivity and reliability is challenging, which is important for large scale manufacturing. In the present study, we produced the two-component HPV B19 VLPs using the *Hansenula polymorpha* expression platform developed by our laboratory in which two plasmids respectively encoding VP1 and VP2 were constructed to co-express the target proteins. The relative amount of VP1 to VP2 was regulated by changing the protomers, and the recombinant yeast strains were screened to optimize the expression level with a stable VP1:VP2 ratio. Our expression platform has the advantage of low cost, which is practical for large scale manufacturing. The study results show that the VLPs produced by *Hansenula polymorpha* are efficiently expressed, highly purified, morphologically homogenous, and good immunogenic, which may serve as promising antigen candidates for developing VLP-based vaccines. 

HPV B19 natural virions are composed of approximately 5% VP1 and 95% VP2. We produced the two-component VLP with the similar composition to natural virions (i.e., VP1/VP2 VLP) using *Hansenula polymorpha*. Immunogenicity evaluation showed that the containing of ~5% VP1 in the VLP significantly improved the IgG antibody response but did not distinctly enhance the neutralizing antibody level. Then, the relative amount of VP1 in the VLP was improved to ~25% by using a stronger protomer (i.e., VP1h/VP2 VLP). It was found that the VLP with this higher content of VP1 exhibited significantly improved neutralizing antibody response. These results are consistent with other studies [18,19]. Our results indicate that it is necessary for vaccine development to use the VLP, with a higher VP1:VP2 ratio than naturally occurring one, as the antigen. 

It has been revealed that VP1u plays a crucial role in eliciting immune responses against HPV B19. However, the location of VP1u in the capsid is not clear. Two seemingly conflicting results have been obtained by immunological experiments. In the recombinant VLPs expressed by insect cells, VP1u was found to be exposed on the capsid surface [17,22], whereas in the natural virus, it was detected to be inside the capsid [23]. In the present study, the location of the VP1u in our recombinant HPV B19 VLPs was determined by (IGEM), and the results indicate that VP1u may be located inside the capsid. It should be pointed out that the IGEM experiment provided preliminary evidence for the internal location of VP1u, and it should be further verified by more lines of evidence in the future. Several studies have indicated that VP1u plays critical roles for HPV B19 infection to host cells [32,33], and it becomes exposed upon heating, low-pH treatment, or receptor attachment [23,24]. The similar phenomenon has also been observed in other parvoviruses [34,35]. For the VLPs produced by *Hansenula polymorpha*, it is not known whether VP1u could be exposed from the capsid by changing expression, purification or environment conditions, which will be investigated in the follow-up studies.

## 5. Conclusions

HPV B19 is a highly infectious virus that can result in serious consequences for the fetus and the immunocompromised persons. At present, no approved vaccine or drug is available against the virus and thus the development of effective vaccines is needed. HPV B19 cannot yet be cultured in vitro, and the recombinant VLPs are promising candidates for vaccine design. In the present work, by using the *Hansenula polymorpha* expression system developed by our laboratory, the VP1 and VP2 were co-expressed to produce the two-component HPV B19 VLPs. Furthermore, the promoter for the expression of VP1 was optimized to improve the amount of VP1 in the VLPs. The recombinant VLPs produced by *Hansenula polymorpha* exhibit high concentrations, morphological homogeneity, and good immunogenic effects. It is also found that the containing of VP1 and increasing its content are important for the recombinant VLPs to elicit high-level neutralizing antibodies against HPV B19. Moreover, IGEM observations indicate that the VP1u with predominant neutralizing activity may be located inside the capsid, similar to that in the capsid of a natural virus. In addition, our expression system has the property of low cost. Our studies provide a potential candidate and an efficient expression system for the development of anti-HPV B19 vaccines.

## Data Availability

Not applicable.
